# HACE1 (HECT domain and ankyrin repeat containing E3 ubiquitin protein ligase 1)

**DOI:** 10.4267/2042/49701

**Published:** 2012-12

**Authors:** Feng Cui, Yanzhuang Wang

**Affiliations:** Department of Molecular, Cellular and Developmental Biology, University of Michigan, Ann Arbor, MI 48109-1048, USA (FC, YW)

## Identity

**HGNC (Hugo):** HACE1

**Location:** 6q16.3

## DNA/RNA

### Note

HACE1 was first cloned by sequencing clones obtained from a fetal brain cDNA library, and was designated as KIAA1320 (Nagase et al., 2000).

The cDNA encodes a 909 amino acid protein (GenBank: NM_020771.3).

### Description

The HACE1 gene is located on chromosome 6 (105175968–105307794); 24 exons.

## Protein

### Description

Human HACE1 encodes HACE1 protein, which containing 909 amino acid residues (size ~103 kDa) (Anglesio et al., 2004). HACE1 possesses six N-terminal ankyrin repeats (amino acid: 64–93; 97–126; 130–159; 163–192; 196–227; 228–257) and a C-terminal HECT domain (amino acid: 572–909; active cysteine residue at position 876).

### Expression

Anglesio et al. showed that a 4,6-kb transcript of HACE1 with strong expression in heart, brain, placenta, pancreas, as well as adult and fetal kidney. HACE1 expression is virtually undetectable in the SK-NEP-1 Wilms’ tumor cell line and in four of five additional primary Wilms’ tumor cases compared with patient-matched normal kidney (Anglesio et al., 2004). Zhang et al. showed that HACE1 mRNA transcripts are ubiquitously expressed in normal human tissues; but the mRNA expression of HACE1 decreases markedly in breast, renal, thyroid, vulva and liver tumors compared to the organ-matched normal tissue samples from the same individuals.

The transcripts are downregulated in almost all lines from the NCI-60 panel of human cancer cell lines compared to control HEK293 cells.

Lower expression is strongly associated with hypermethylation of two CpG islands located upstream of the HACE1 locus (Zhang et al., 2007).

HACE1 is also downregulated in natural killer/T-cell lymphoma of the nasal type (NKTCL) (Huang et al., 2010), colorectal cancer (Hibi et al., 2008) and gastric cancer (Sakata et al., 2009). However, the expression of HACE1 varies in different breast cancer cell lines (Kao et al., 2009). In neuroblastomas, low HACE1 expression is associated with worse overall survival, suggesting that HACE1 may function as a tumor suppressor (Diskin et al., 2012).

### Localisation

Anglesio et al. indicated that the subcellular localization of HACE1 is predominantly in the endoplasmic reticulum (ER) and cytosol, and a small amount of endogenous protein is also present in other fractions of NIH3T3 cells (Anglesio et al., 2004). Tang et al. showed that a fraction of HACE1 colocalizes with Golgi markers in HeLa and normal rat kidney (NRK) cells; the association of HACE1 with Golgi membranes is through interaction with Golgi Rab GTPases, in particular Rab1 (Tang et al., 2011).

### Function

#### Tumor suppressor

Anglesio et al. showed that HACE1 possesses intrinsic ubiquitin ligase activity, utilizing UBCH7 as a candidate partner E2 enzyme (Anglesio et al., 2004). Zhang et al. demonstrated that HACE1 is frequently downregulated in human tumors and maps to a region of chromosome 6q21 implicated in multiple human cancers. Genetic inactivation of HACE1 in mice resulted in the development of spontaneous, late-onset cancer. Their data suggested that HACE1 is involved in phosphorylation dependent degradation of cyclin D1, indicating a role for HACE1 in inhibiting cell cycle progression (Zhang et al., 2007).

HACE1 deficient mice are spontaneously prone to developing multiple malignant tumors in various organs (Zhang et al., 2007).

#### Degradation of Rac1

In 2011, Torrino et al. reported that HACE1 binds preferentially GTP-bound Rac1 and catalyzes its polyubiquitination. HACE1 expression increases the ubiquitination of Rac1 when the GTPase is activated. HACE1 is required for cytotoxic necrosis factor 1 (CNSF1)-mediated depletion of Rac1 and efficient invasion of endothelial cell monolayer by bacteria, suggesting that HACE1 plays a major role in host defense against pathogens (Torrino et al., 2011). Castillo-Lluva et al. further showed that HACE1 catalyzes polyubiquitination of Rac1 at lysine 147 following its activation by a migration stimulus, such as hepatocyte growth factor (HGF), resulting in Rac1 degradation by the proteasome. HACE1-depletion is accompanied by an increased total Rac1 level and accumulation of Rac1 in membrane ruffles.

Furthermore, HACE1-depletion leads to enhanced cell migration, which may be significant for malignant conversion (Castillo-Lluva et al., 2012).

#### Golgi biogenesis

Tang et al. reported that HACE1 is targeted to the Golgi membrane through interactions with Rab proteins. The ubiquitin ligase activity of HACE1 in mitotic Golgi disassembly is required for subsequent post-mitotic Golgi membrane fusion. Depletion of HACE1 using small hairpin RNAs or expression of an inactive HACE1 mutant protein in cells impairs post-mitotic Golgi membrane fusion. The identification of HACE1 as a Golgi-localized ubiquitin ligase provides evidence that ubiquitin has a critical role in Golgi biogenesis during the cell cycle (Tang et al., 2011).

#### Repression of the RAR (retinoic acid receptor)-regulated transcription

HACE1 was isolated as a RARβ3 AB region interacting protein. HACE1 functionally represses the transcriptional activity of RARα1 and RARβ isoforms 1, 2 and 3, but not RARγ1 in luciferase reporter assays. In addition, HACE1 represses several endogenous RAR-regulated genes. The E3 ubiquitin ligase activity is not required for the repression effect of HACE1 on the transcriptional activity of RARβ3. HACE1 also inhibits the retinoic acid-dependent degradation of RARβ3. The repression of RAR-regulated transcription by HACE1 is possibly due to its ability to inhibit RA-induced degradation of RARs (Zhao et al., 2009).

#### Determinant of the equol-producing phenotype

HACE1 was identified in a genome-wide association study (GWAS) designed to find genetic factors associated with the equol-producing phenotype in Korean population.

The authors identified 5 single-nucleotide polymorphisms in HACE1. Individuals with a minor allele of the most significant SNP rs6927608 did not produce equol.

The interaction between equol production and the rs6927608 HACE1 SNP was significantly associated with systolic blood pressure. Finally, the authors concluded that equol production is linked to blood pressure, and HACE1 might be a determinant of the equol-producing phenotype (Hong et al., 2012).

### Homology

The HACE1 gene is conserved in chimpanzee, dog, cow, mouse, rat, chicken, and zebrafish.

## Implicated in

### Sporadic Wilms’ tumor

#### Note

In 2004, Anglesio et al. analyzed the chromosome 6q21 breakpoint of a non-constitutional t(6;15)(q21;q21) rearrangement in sporadic Wilms’ tumor. They showed that although the HACE1 locus was not directly interrupted by the translocation in the index Wilms’ case, its expression was markedly lower in tumor tissues compared with adjacent normal kidney. Moreover, HACE1 expression is virtually undetectable in the SK-NEP-1 Wilms’ tumor cell line and in four of five additional primary Wilms’ tumor cases compared with patient-matched normal kidney. Their data implicated that low expression of HACE1 was associated with sporadic Wilms’ tumor (Anglesio et al., 2004).

In 2010, Slade et al. indentified a t(5;6)(q21;q21) translocation in a child with bilateral, young-onset Wilms’ tumor. The 6q21 breakpoint transects and truncates HACE1, which has been implicated as a somatically inactivated target in Wilms tumorigenesis. To evaluate the contribution of HACE1 to Wilms’ tumor predisposition, the gene was screened in 450 individuals with Wilms’ tumor. One child with unilateral Wilms’ tumor and a truncated HACE1 mutation was identified. It was concluded that constitutional disruption of HACE1 likely predisposes Wilms’ tumor. However, HACE1 mutations are rare and therefore can only make a small contribution to Wilms’ tumor incidence (Slade et al., 2010).

### Lymphoma

#### Note

HACE1 is downregulated in natural killer/T-cell lymphoma of the nasal type (NKTCL) in both gene expression profiling and array-base comparative genomic hybridization analyses. Since HACE1 is the target of epigenetic inactivation in Wilms’ tumor and has been proposed as a tumor suppressor gene in multiple human cancers, it is tempting to speculate that HACE1 might be involved in the pathogenesis of NKTCL (Huang et al., 2010).

### Colorectal carcinomas and gastric cancer

#### Note

The methylation status of the HACE1 gene was examined in primary carcinomas and the corresponding normal tissues derived from 32 patients with colorectal cancer using quantitative methylation-specific PCR (qMSP), and the correlation between the methylation status and the clinic pathological findings was evaluated. The aberrant methylation of HACE1 was frequently observed in colorectal cancer. Thus HACE1 might act as a tumor suppressor in colorectal carcinomas and HACE1 methylation might present a malignant potential in colorectal cancer (Hibi et al., 2008). Similar research was performed in primary gastric carcinomas, and HACE1 was found frequently methylated in gastric carcinoma derived from male patients (Sakata et al., 2009).

### Breast cancer

#### Note

Kao et al. utilized the whole-genome DNA microarrays to profile gene expression and DNA copy number alterations (CANs) in a collection of 52 widely used breast cancer cell lines, and comparisons were made to existing profiles of primary breast tumors. The expression level of HACE1 varies between different breast cancer cell lines. The contribution of HACE1 breast cancer biogenesis is still unclear (Kao et al., 2009).

### Neuroblastomas

#### Note

In a genome-wide association study of 2817 neuroblastoma cases and 7473 controls, HACE1 and LIN28B were identified as two new associations at 6q16.

Low HACE1 and high LIN28B expression in diagnostic primary neuroblastomas are associated with worse overall survival. These data suggested that HACE1 might function as a tumor suppressor and LIN28B as an oncogene in advanced neuroblastomas (Diskin et al., 2012).

## Figures and Tables

**Figure:**
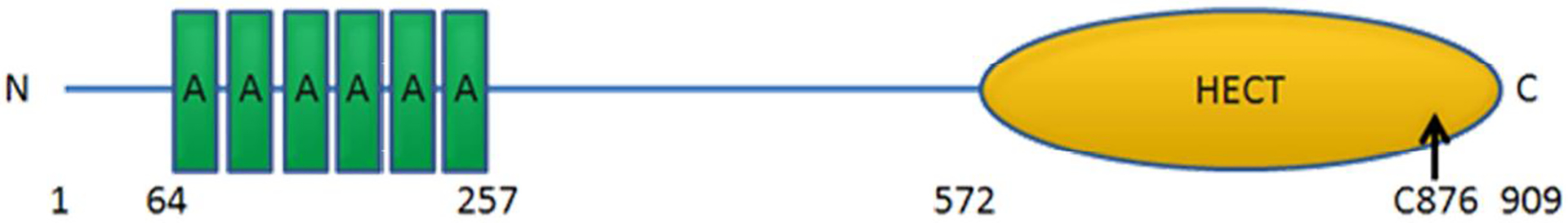
Schematic structure of the HACE1 protein. HACE1 contains six ankyrin repeats at the N terminus and a HECT domain at the C terminus. A cysteine residue essential for the ubiquitin ligase activity is at position 876.
